# Horticultural applications of natural hybrids as an accelerating way for breeding woody ornamental plants

**DOI:** 10.3389/fgene.2022.1047100

**Published:** 2022-11-09

**Authors:** Xiao-Ling Tian, Yong-Peng Ma

**Affiliations:** ^1^ Guiyang Institute of Humanities and Technology, Guiyang, China; ^2^ Yunnan Key Laboratory for Integrative Conservation of Plant Species With Extremely Small Populations, Kunming Institute of Botany, Chinese Academy of Sciences, Kunming, China

**Keywords:** hybrid swarm, natural hybrids, woody ornamental plants, rhododendron (Ericaceae), SNP data

China has abundant woody ornamental plant resources comprising a large diversity of families, genera, and species in the wild. There is also a great demand for woody ornamental plants on the Chinese market. It has been estimated that China has the largest production area worldwide (740,954 ha) for the production of woody ornamentals, with a value of 8051 million EUR (AIPH, 2015). Despite Chinese traditional woody cultivars (e.g., roses, *Osmanthus*, and *Camellias*), imported woody ornamental plants have become dominant on the Chinese horticulture market. It should be noted that, in this study, we exclude any woody ornamental plants found on the market without accurate ancestral and/or breeding information, such as many azaleas on the Chinese market (Chang et al., 2020). In contrast to the large diversity of wild resources, horticultural applications of woody ornamental plants remain insufficient, due to long vegetative cycles, making breeding challenging. Moreover, the unavailability of whole-genome information and the difficulties in establishing genetic transformation systems for most woody ornamental plants mean that potential applications of genome editing in these plants have also been limited at present (Van Laere et al., 2018).

Most existing frameworks for breeding of woody ornamental plants have focused on creation of new morphological variations, like classical/traditional cross breeding and artificial polyploidization (Van Huylenbroeck and Van Laere, 2008). Currently, there are few options to accelerate the breeding process, particularly for woody plants with long vegetative cycles. We therefore raise natural hybrids as valuable candidates for solving the problem and facilitating selection of new cultivars.

It is well recognized that natural hybridization occurs frequently in >25% of known plant species (Mallet, 2005) and plays important roles in several aspects of evolution, including the origins of new ecotypes or species, the origin and transfer of genetic adaptations, and the reinforcement or breakdown of reproductive barriers (Rieseberg and Carney, 1998). Due to the characteristics of different genetic combinations from parental species in natural hybrids, various phenotypes for horticultural usage can be created, including different leaf types, flower shapes, colors, and scents. However, this great potential of natural hybrids to act as a source for the breeding of new cultivars has been often ignored, probably owing to the big gap in focus between plant taxonomists and horticulturalists worldwide.

Whether these natural hybrids are a stable, long-term phenomenon or a relatively recent occurrence, certain hybrid genotypes will have been selected out because of genetic incompatibilities and other reasons (e.g., the BDM incompatibility model, Coyne and Orr, 2004). Thus, those natural hybrids with poor adaptability are effectively removed by nature and the remaining hybrids that we find growing in the field may be adapted to the local environment. With respect to this aspect, using natural hybrids for the breeding of new cultivars is not only time saving but may also result in fitter and more locally adapted cultivars than those created by traditional open or controlled cross-pollinations, which can often produce unfit hybrids due to hybrid sterility and/or inviability.

**FIGURE 1:**
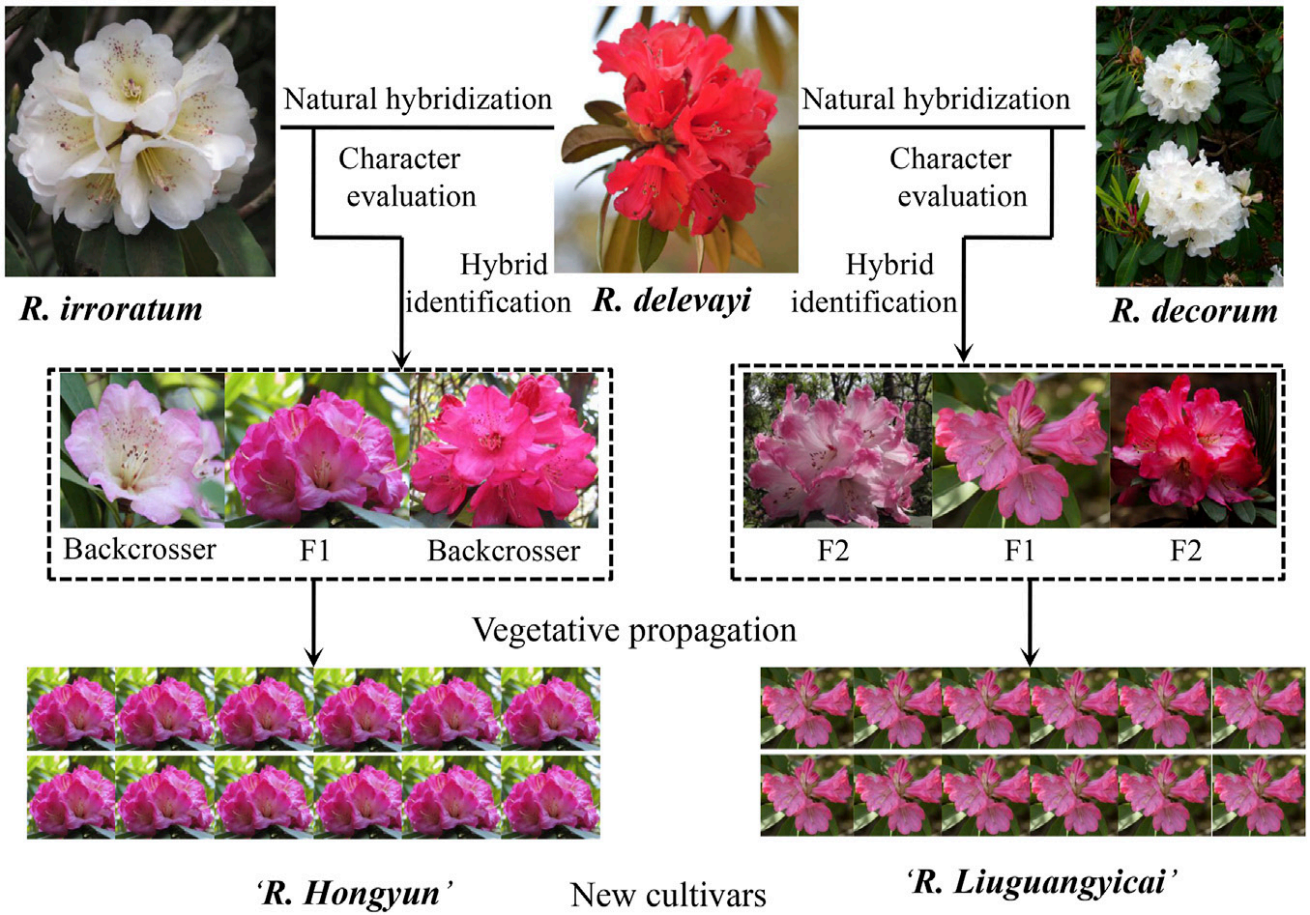
Framework with a step-by-step approach for the rapid production of new cultivars from natural hybrids, using case studies from alpine rhododendrons.

Here, we develop a framework with a step-by-step approach for the rapid production of new cultivars from natural hybrids (Figure 1). We use case studies from alpine rhododendrons, which have a much higher horticultural value than those of azaleas (e.g., larger leaves, improved floral shapes, scents, and various colors), but which take a longer time than azaleas to flower (some azaleas can flower from seedlings within 2 years). 1) Confirmation of natural hybrids by integrating morphological and genetic evidence. For early generation hybrids, intermediate morphological characteristics are often displayed (Ma et al., 2010a,b; Ma et al., 2014), and we have demonstrated that most natural hybrids between *Rhododendron delavayi* and *R. irroratum* in the Baili Scenic Reserve (in the western Guizhou province) seem to have been detectable with adequate morphological data alone (detailed statistical analysis of morphological data can be found in Marczewski et al., 2016). Further confirmation of hybrids can be carried out using genetic data (e.g., ISSR, AFLP, SSR, and SNPs). For example, principal component analysis (PCA) implemented in GenAlEx 6.0 (Peakall and Smouse, 2006) and structural analysis of genetic data can reveal genetic relationships between hybrids and parents (Hubisz et al., 2009), and the genetic compositions of hybrids from parental species, whereas NewHybrids (Anderson and Thompson, 2002), together with data simulations, can clarify six genotypes (two parents, F_1_, F_2_, and two backcrosses to F_1_) to which natural hybrids may belong (Ma et al., 2014, 2019). If sufficient molecular markers (e.g., SNP data) can be obtained, up to 45 natural hybrid genotypes can be assessed (Milne and Abbott, 2008). 2) Characteristic evaluation and introduction of natural hybrids into botanical gardens or nurseries. Through this step, natural hybrids with good ornamental value and adaptations can be targeted. However, the horticultural success of these natural hybrids also depends on their survival and easy vegetative propagation in alternate habitats. Some work with regards to the introduction, and domestication of these natural hybrids into a horticultural environment needs to be performed. 3) Vegetative propagation to obtain uniformity and stability in the horticultural characteristics of natural hybrids. In general, stability in horticultural characteristics can be achieved through rooting and grafting; although if these methods are unsuccessful, tissue culture can be employed to fix the ornamental characteristics. 4) New cultivar application following guidelines of the DUS (distinctness, uniformity, and stability) test and subsequent commercial demonstrations.

Although in the genomic era, integration of the genomic technologies (e.g. GWAS associated trait selection) for ornamental woods with availability of whole-genome information will enhance selection efficiency, we are confident that our framework can provide a reference and be beneficial to colleagues, particularly those working toward breeding new cultivars of most woody ornamental plants without whole-genome information and establishment of genetic transformation systems. Several new cultivars of Chinese indigenous plants generated according to our proposed framework will be emerging in the near future, and we hope that some of them will be commercially competitive against the imported cultivars on the Chinese flower market even before the successful application of genome editing in woody ornamental plants in China.
